# Potential biomarker proteins for aspiration pneumonia detected by shotgun proteomics using buccal mucosa samples: a cross-sectional case–control study

**DOI:** 10.1186/s12014-023-09398-w

**Published:** 2023-03-09

**Authors:** Kohei Ogura, Maho Endo, Takashi Hase, Hitomi Negami, Kohsuke Tsuchiya, Takumi Nishiuchi, Takeshi Suzuki, Kazuhiro Ogai, Hiromi Sanada, Shigefumi Okamoto, Junko Sugama

**Affiliations:** 1 Advanced Health Care Science Research Unit, Institute for Frontier Science Initiative, Kanazawa University, 5-11-80 Kodatsuno, Kanazawa, Ishikawa 9200942 Japan; 2 Nursing Department, Fujita Health University Hospital, 1-98 Dengakugakubo, Kutsukake-Cho, Toyoake, Aichi 4701192 Japan; 3 Department of Oral and Maxillofacial Surgery, Noto General Hospital, 6-4 Fujibashi, Nanao, Ishikawa 9260816 Japan; 4 Division of Immunology and Molecular Biology, Cancer Research Institute, Kanazawa University. Kakuma-Cho, Kanazawa, Ishikawa 9201164 Japan; 5 Division of Functional Genomics, Advanced Science Research Center, Kanazawa University, Kanazawa, Ishikawa 9200934 Japan; 6 Division of Functional Genomics, Cancer Research Institute, Kanazawa University. Kakuma-Cho, Kanazawa, Ishikawa 9201164 Japan; 7 Institute of Medical, Pharmaceutical and Health Sciences, AI Hospital/Macro Signal Dynamics Research and Development Center, Kanazawa University, 5-11-80 Kodatsuno, Kanazawa, Ishikawa 9200942 Japan; 8 Ishikawa Prefectural Nursing University, 1-1 Gakuendai, Kahoku, Ishikawa 929-1210 Japan; 9 Department of Clinical Laboratory Sciences, Faculty of Health Sciences, Institute of Medical, Pharmaceutical, and Health Sciences, Kanazawa University, 5−11-80 Kodatsuno, Kanazawa, Ishikawa 9200942 Japan; 10 Research Center for Implementation Nursing Science Initiative, Innovation Promotion Division, Fujita Health University, 1-98 Dengakugakubo, Kutsukake-Cho, Toyoake, Aichi 4701192 Japan

**Keywords:** Aspiration pneumonia, Biomarker, Proteomics, Buccal mucosa

## Abstract

**Background:**

Aspiration pneumonia (AP), which is a major cause of death in the elderly, does present with typical symptoms in the early stages of onset, thus it is difficult to detect and treat at an early stage. In this study, we identified biomarkers that are useful for the detection of AP and focused on salivary proteins, which may be collected non-invasively. Because expectorating saliva is often difficult for elderly people, we collected salivary proteins from the buccal mucosa.

**Methods:**

We collected samples from the buccal mucosa of six patients with AP and six control patients (no AP) in an acute-care hospital. Following protein precipitation using trichloroacetic acid and washing with acetone, the samples were analyzed by liquid chromatography and tandem mass spectrometry (LC–MS/MS). We also determined the levels of cytokines and chemokines in non-precipitated samples from buccal mucosa.

**Results:**

Comparative quantitative analysis of LC–MS/MS spectra revealed 55 highly (*P* values < 0.10) abundant proteins with high FDR confidence (*q* values < 0.01) and high coverage (> 50%) in the AP group compared with the control group. Among the 55 proteins, the protein abundances of four proteins (protein S100-A7A, eukaryotic translation initiation factor 1, Serpin B4, and peptidoglycan recognition protein 1) in the AP group showed a negative correlation with the time post-onset; these proteins are promising AP biomarker candidates. In addition, the abundance of C-reactive protein (CRP) in oral samples was highly correlated with serum CRP levels, suggesting that oral CRP levels may be used as a surrogate to predict serum CRP in AP patients. A multiplex cytokine/chemokine assay revealed that MCP-1 tended to be low, indicating unresponsiveness of MCP-1 and its downstream immune pathways in AP.

**Conclusion:**

Our findings suggest that oral salivary proteins, which are obtained non-invasively, can be utilized for the detection of AP.

**Supplementary Information:**

The online version contains supplementary material available at 10.1186/s12014-023-09398-w.

## Background

Aspiration pneumonia (AP) is caused by inhaling saliva, food, or vomit, which results in bacterial infection [1–3]. Aspiration, defined as the inhalation of oropharyngeal or gastric contents into the larynx and lower respiratory tract, is often the result of impaired swallowing resulting from dysphagia, head/neck/esophageal cancers, esophageal stricture, chronic obstructive pulmonary disease, or seizures. This allows oral and/or gastric contents to enter the lung, especially in patients with an inefficient cough reflex [1]. In addition to swallowing, impaired consciousness, because of degenerative neurologic disease or cardiac arrest, is also a risk factor for AP [2, 4]

Patients with bacterial AP need to be treated promptly with antibiotics. Delay in diagnosis and treatment can result in prolonged hospital stay, additional complications, and eventually death [5]. However, pneumonia symptoms such as cough and fever often do not appear in the early stages. This absence of symptoms restricts the early detection and treatment of AP. Although the detection of causative bacteria results in prompt treatment with antibiotics, it is often difficult to distinguish infectious and noninfectious oral bacteria. Oral bacteria are present at various sites within the human oral cavity [6]. Recent reports have indicated that lung microbiota is involved in pneumonia in addition to the oral microbiota [7, 8]. Boaden et al. identified 103 different bacterial phylotypes from the oral microbiota of patients with acute stroke [9]. One study identified 67 pathogens in 95 institutionalized elderly patients with severe AP [4]. These reports indicate that host immune system-derived biomarkers, but not causative bacteria, are useful for detecting AP in the early stage.

It is unclear whether AP represents a distinct entity from typical pneumonia [1, 10, 11]. Based on a previous report which estimated that AP accounts 5%–15% of the cases of community-acquired pneumonia, Mandell et al. proposed that AP should not be considered a distinct entity, but rather part of a continuum that also includes community- and hospital-acquired cases of pneumonia [1]. Recent studies have indicated that the composition of salivary proteins reflects oral and systemic conditions [12]. For example, salivary proteins may apply to the detection of localized oral diseases, such as head and neck cancer [13] and Sjogren's syndrome [14, 15], as well as systemic diseases, such as diabetes mellitus [16–18], and viral infections [19]. Based on reports regarding a relationship between oral proteomics and disease, we suspect that some salivary proteins may be used as biomarkers for the detection of AP at the early stage of onset.

It is not easy for elderly people, particularly bedridden patients with neurologic or cerebrovascular disease, to eject a sufficient volume of saliva. In addition, saliva production is likely decreased because of decreased chewing frequency or drug treatment. In the present study, we collected saliva proteins from the buccal mucosa, where the ostia of Stensen’s ducts are located. By cleaning the buccal mucosa before sample collection, contaminates are readily removed. Using shotgun proteomics for a comparative quantitative analysis between AP and control patients, we identified eight candidate AP biomarkers. We also found a significant correlation between serum and oral C-reactive protein (CRP) levels. In addition, we evaluated a panel of cytokines and chemokines to determine the responsiveness of immune-related proteins.

## Methods

### Subjects

We collected samples from the buccal mucosa of six AP and six control patients in an acute-care hospital in Ishikawa Prefecture (Japan) from September 2021 to December 2021. The characteristics of the 12 patients are listed in Tables 1 and Additional file 1: Table S1. Five of the six AP patients and one of the six controls had been treated with antimicrobial agents at the time of collection. Five of the AP patients had a history of AP. Because we selected the six patients who had never been previously diagnosed with AP as controls, this study is a cross-sectional case–control study. Medical information (age, gender, body mass index, underlying diseases, blood test data, and dietary intake method) was obtained from the electronic medical records. Oral conditions were assessed by the Oral Health Assessment Tool (OHAT) [20]. The number of remaining teeth and the presence of intra-oral bleeding were determined and the buccal mucosa was assessed for dryness.

**Table 1:** Characteristics of the subjects

	AP (*n* = 6)	Ctrl (*n* = 6)	*P*-value^†^
Age (years)	87.2 ± 5.5	78.3 ± 10.3	0.132
Body Mass Index	15.3 ± 0.2	21.6 ± 3.6	0.002
Blood data			
CRP (mg/dl)	4.99 ± 4.27	1.80 ± 2.86	0.132
WBC (10^3^/µl)	8.07 ± 3.58	8.57 ± 4.30	1.000
Alb (g/dl)^*^	2.78 ± 0.59	2.80 ± 1.62	1.000
Oral states			
Residual teeth	6.8 ± 8.5	8.2 ± 12.8	0.818
OHAT	3.2 ± 2.2	1.2 ± 1.2	0.093

^†^*P* values were calculated by Mann–Whitney U test

^*^Albumin concentrations of the AP (*n* = 4) and control (*n* = 4) patients. The other data were obtained from the AP (*n* = 6) and control (*n* = 6) patients

### Sample collection from the buccal mucosa

Before sample collection, a dentist confirmed that at least 2 h had passed since the previous meal. After removing visible food residue, samples were collected from the buccal mucosa using a Hummingood sponge brush (Molten Corporation, Hiroshima, Japan), which had been dipped into 5 mL saline in a 50 mL tube and squeezed briefly onto the side of the tube. The samples were collected by placing the brush on the buccal mucosa, rubbing “back and forth” 10 times at a rate of 1 rub/second. The sponge was returned to the saline-containing tube, pressed, and squeezed tightly. After collection, the samples were stored at − 20 °C, thawed, and centrifuged at 3,000 × g for 5 min at 4 °C. The supernatants were used for further analysis by LC–MS/MS and multiplex assays.

### LC–MS/MS

The supernatant was precipitated using trichloroacetic acid (TCA) and washed with acetone. The precipitate was air-dried at room temperature and dissolved in 40 μL of 6 M urea containing 50 mM triethylammonium bicarbonate. After measuring the protein concentration using Pierce BCA Protein Assay Kit (ThermoFisher Scientific), 1 μg of protein was incubated with 5 mM tris(2-carboxyethyl)phosphine) for 30 min at 37 °C under dark conditions, alkylated with 24 mM iodoacetamide for 30 min at room temperature, and digested with trypsin (Promega) at a trypsin: protein ratio of 1:10. After desalination using Pierce C18 Spin Tips & Columns (ThermoFisher Scientific) and acidification with 1% trifluoroacetic acid, the digested peptides were loaded onto the nanoliquid chromatography EASY-nLC 1200 system (ThermoFisher Scientific). This system is equipped with a precolumn (Acclaim PepMap100 C18 column: inner diameter, 75 μm, length, 20 mm, particle size, 3.0 µm; ThermoFisher Scientific) and analytical column (Acclaim PepMap100 C18 column: inner diameter, 75 μm; length, 150 mm, particle size, 3.0 µm; ThermoFisher Scientific) equilibrated with 0.1% formic acid. Next, peptide elution is performed using a linear gradient (0%–35%) of acetonitrile at a flow rate of 300 mL/min. The eluted peptides were ionized with a spray voltage of 2 kV (ion transfer tube temperature, 275 °C) and detected using tandem mass spectrometry (LC–MS/MS; Thermo Orbitrap QE plus, ThermoFisher Scientific) in the data-dependent acquisition mode using Xcalibur (version 4.0; Thermo Fisher Scientific). Mass spectra with 375–1,500 m/z were obtained with a resolution of 70,000 full width at half maximum.

### Quantification of LC–MS/MS data

Comparative analysis of the detected protein and label-free quantitation was performed using Proteome Discoverer software version 2.2.0.388 (Thermo Fisher Scientific). The proteins were searched against UniProtKB/Swiss-Prot human database (taxonomy_id:9606). Oxidation of methionines and carbamidomethylation of cysteines were set as variable and fixed modification, respectively. The mass tolerance was set to 10 ppm. Two missed cleavages by trypsin were permitted. Further, target-decoy approach was used to determine the false discovery rate (FDR). Peptide-to-spectrum match data were obtained at an FDR of 1%, and the abundances were normalized by total peptide amounts.

### Multiplex cytokine assay

After measuring protein concentration using the BCA Protein Assay Kit, the supernatants (not precipitated by TCA) were applied to a LEGENDplex Human Inflammation Panel 1 (13-plex: IL-1β, IFN-α2, IFN-γ, TNF-α, MCP-1, IL-6, IL-8, IL-10, IL-12p70, IL-17A, IL-18, IL-23, IL-33) in a V-bottom Plate (BioLegend, San Diego, USA). The concentrations of the target proteins were standardized to total protein concentration.

## Results

### Sample collection and patient information

We collected buccal mucosa samples from AP (*n* = 6, age 79–93 years old) and non-AP (Control; *n* = 6, age 66–93 years old) patients. Three samples (AP#3, AP#5, and AP#6) were collected from the patients with Parkinson’s disease, three (AP#2, AP#4, and Control#1) from those with Alzheimer's disease, and two (Control#2 and Control#6) with Lacunar infarction. The duration from the onset to the collection time varied from 1 to 18 days (Additional file 1: Table S1). Body mass index was low in the AP group (Table 1). No significant differences were observed in the blood for CRP concentration, several white blood cells, or serum albumin concentration. While there was no difference in the number of residual teeth, the OHAT score tended to be higher in the AP group, indicating an unhealthy oral state of the AP patients.

### Comparative quantitative analysis of oral proteins

After TCA precipitation and acetone wash, protein solutions were obtained with concentrations ranging from 0.65 to 2.49 mg/mL. LC–MS/MS analysis detected 3,528 proteins including 3,253, 157, and 118 proteins at high- (*q* value < 0.01), middle- (0.01 < q value < 0.05), and low-confidence levels, respectively, based on their FDR (Additional file 2: Figure S1 and Additional file 1: Table S2). No significant difference was observed in the abundance distribution (Additional file 2: Figure S2). Principal component analysis (PCA) revealed that AP #4 was distinct from the other 11 samples; there was no significant difference in PCA profiles of the AP and control groups (Additional file 2: Figure S3). Although Proteome Discoverer software version 2.2, which applies the Minora nodes, is a powerful tool for label-free quantification [21], ANOVA adjusted using the Benjamini–Hochberg method, and not using nonparametric analysis such as Mann–Whitney U test, is available to calculate *P* values (Additional file 1: Table S2). In the present study, we compared the protein abundance obtained using the software. Among the 3253 proteins with high confidence, 638 had high coverage (> 50%); of those, 601 proteins were detected in all 12 samples. Then, we compared protein abundances of the 601 proteins between the AP and control groups. Abundance of 18 proteins, including aldo–keto reductase family 1 member B10, interleukin-36 receptor antagonist protein, and caspase-14, were significantly high in the AP group (P < 0.05 by Mann–Whitney U test) (Additional file 1: Table S3). Further, 37 proteins, including chloride intracellular channel protein 3, protein S100-A7A, and serpin B4, had high abundance in the AP group (0.1 > *P* ≥ 0.05 by Mann–Whitney U test; Additional file 1: Table S3). Next, we performed the gene ontology enrichment analysis on the proteins with significantly high abundance in the AP group [22, 23]. Significant results (*P* < 0.05 and FDR < 0.05) were observed for three processes: nitrobenzene metabolic process, peptide antigen assembly with MHC class I protein complex, and cellular detoxification of nitrogen compound.

### Serum and oral C-reactive protein

**Fig. 1:**
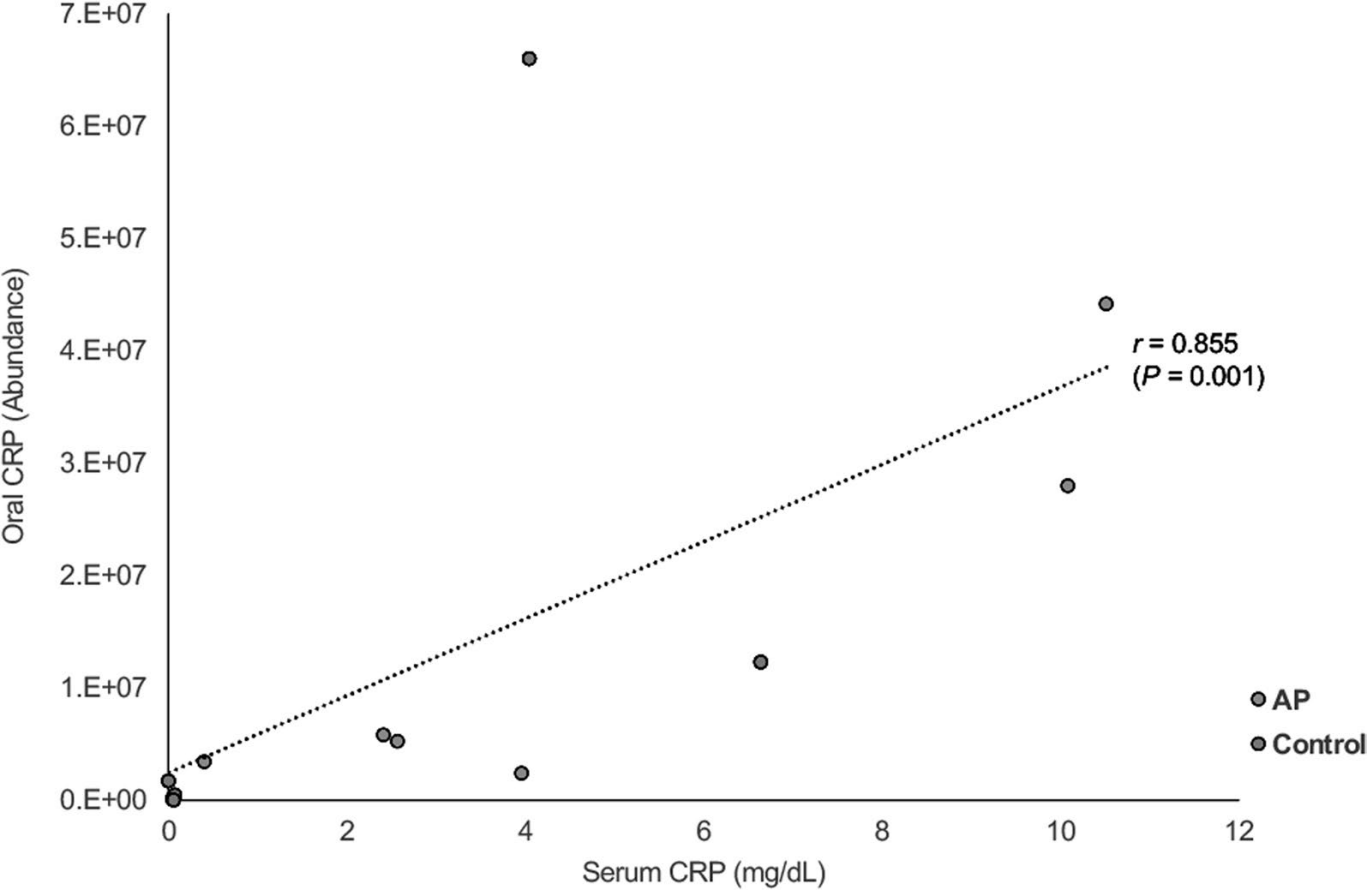
Correlation between serum and oral CRP levels (Spearman's rank correlation coefficient)

Ouellet-Morin et al. reported a moderate-to-strong association between CRP measured in saliva and serum (*r* = 0.72) [24]. In the present study, there was no significant difference in CRP concentration either in the blood (*P* = 0.16) or oral cavity (*P* = 0.71) between the AP and control groups. Nonetheless, Spearman's correlation coefficient was 0.86 (*P* = 0.001) between blood and oral CRP levels, indicating that serum CRP may be predicted non-invasively using oral CRP values, which is consistent with the previous report [24] (Fig. 1). It is unclear why the abundance of oral CRP was relatively high in Control #3.

### Detected proteins

**Fig. 2:**
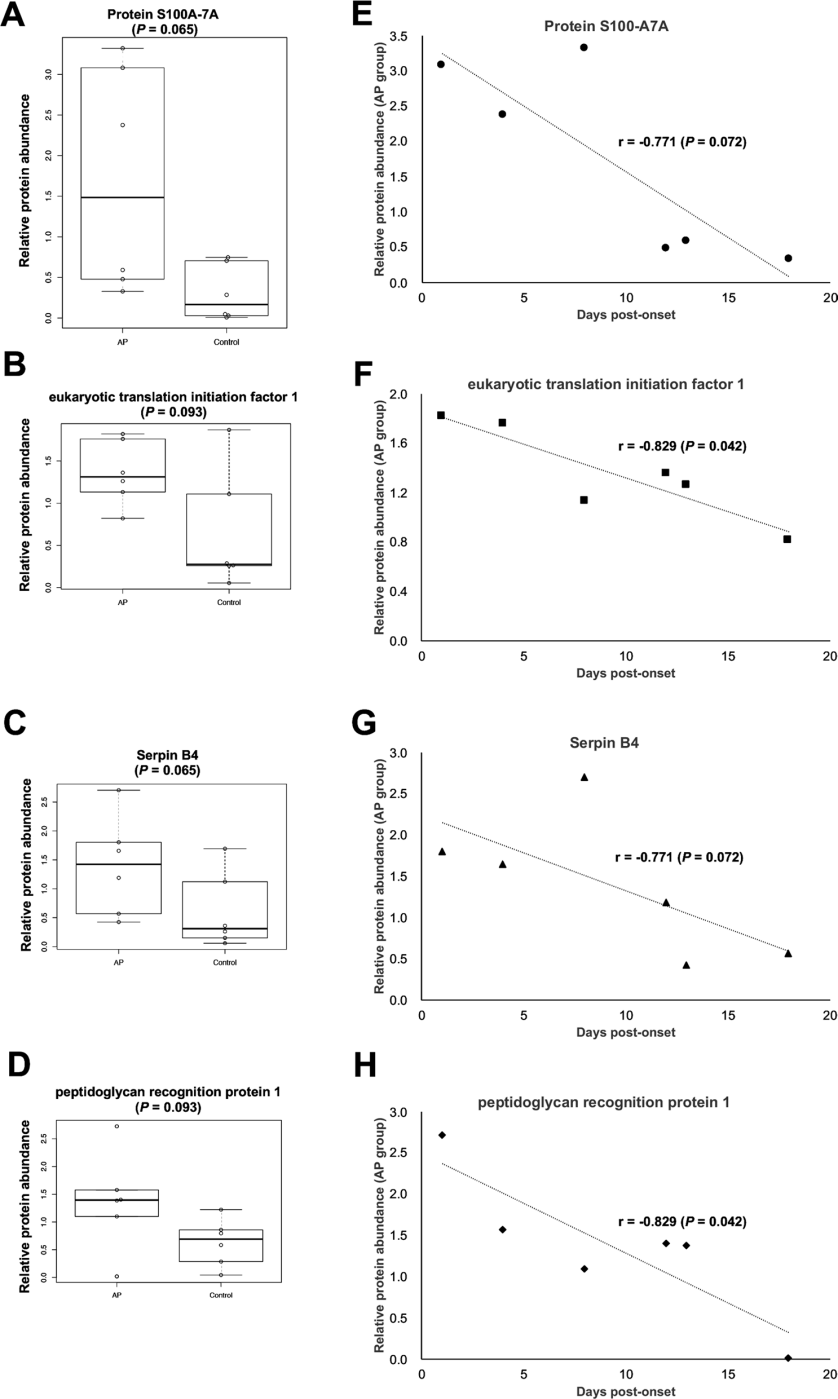
Four proteins expressed at high levels in the AP samples. The proteins detected in all 12 samples are listed in Table 2. The protein abundance between the six AP and six control samples *(A–D)* and the correlation between time post-onset and protein abundance in the six AP samples (*E–H*) are shown

As shown in Additional file 1: Table S1, the time between the day of onset and collection ranged from 1 to 18 days. Next, we examined the correlation between the time from onset (Additional file 1: Table S1) and abundance of the proteins in the AP group (Additional file 1: Table S2). Among the 55 highly expressed proteins (*P* < 0.10 by Mann–Whitney U test) in the AP group, negative correlation was shown by 4 proteins, viz., protein S100-A7A (Uniprot ID, Q86SG5), eukaryotic translation initiation factor 1 (P41567), serpin B4 (P48594), and peptidoglycan recognition protein 1 (O75594) (*P* < 0.10 by Spearman’s test; Table 2 and Fig. 2).

**Table 2:** Proteins with higher abundance between the AP and control groups

Accession	Description	Exp. q-value: Combined	Coverage [%]	Abundance Ratio: (AP)/(Control)^*a^	*P* value^*b^	Correlation coefficient^*c^	*P* value^*d^
Q86SG5	Protein S100-A7A	0	69	6.51	0.065	− 0.771	0.072
P41567	eukaryotic translation initiation factor 1	0	62	3.03	0.093	− 0.829	0.042
P48594	Serpin B4	0	67	2.6	0.065	− 0.771	0.072
O75594	peptidoglycan recognition protein 1	0	53	1.23	0.093	− 0.829	0.042

The proteins were detected in all 12 samples with a coverage of > 50%

^*a^Abundance Ratio was calculated using Proteome Discoverer software version 2.2

^*b^*P* values were calculated by Mann–Whitney U test using the protein abundance (AP vs. Control)

^*c^Correlation coefficients between days post-onset and protein abundance in the AP group

^*d^*P* values were calculated by Spearman’s test

### S100 protein family

The S100 proteins, a family of calcium-binding cytosolic proteins, are known as damage-associated molecular pattern molecules and they exhibit a variety of intracellular and extracellular functions [25]. Protein S100A-7A (Ratio (AP/Control) = 6.51 by Proteins Discoverer software version 2.2) was higher (*P* = 0.065 by Mann–Whitney U test) in the AP group; however, there was no difference in the levels of other S100 protein family members.

### Cytokines and chemokines

The LC–MS/MS detected some interleukins (ILs), however, there was no difference in IL-1α, IL-8, or IL-18 levels between the groups. The IL-36 cytokines, which include IL-36α, IL-36β, IL-36γ and IL-36Ra, belong to the IL-1 family and exert pro-inflammatory effects on various target cells, such as keratinocytes, synoviocytes, dendritic cells, and T cells [26]. Ramadas et al. showed that IL-36γ is upregulated in airway epithelial cells and involved in chemokine (neutrophil chemoattractants CXCL1 and CXCL2) production and neutrophil influx in mice challenged with a house dust mite extract [27]. In contrast, the abundance of the IL-36 receptor antagonist protein was significantly higher in AP samples compared with the control samples (*P* = 0.041). Our data suggest that various IL-36-related signaling pathways are involved in the onset of AP.

### Non-salivary proteins

In the Human Body Fluid Proteome database, 2,871 proteins have been registered as saliva proteins as of May 2022 [28], and 1,973 of the 3,528 proteins detected in the present study were registered as salivary proteins in the database. Of the 1,555 non-salivary proteins, 130 were in high abundance in the AP group, whereas only six were detected with > 20% coverage. Mago Nashi Homolog 2 (Magoh2) was detected in five of the six AP samples (coverage = 39%), but not in any of the Control samples. Although Magoh proteins contribute to exon junction complexes [29], it is unclear whether the Magoh2 protein is involved in the onset of AP.

### Multiplex cytokine assay

**Fig. 3:**
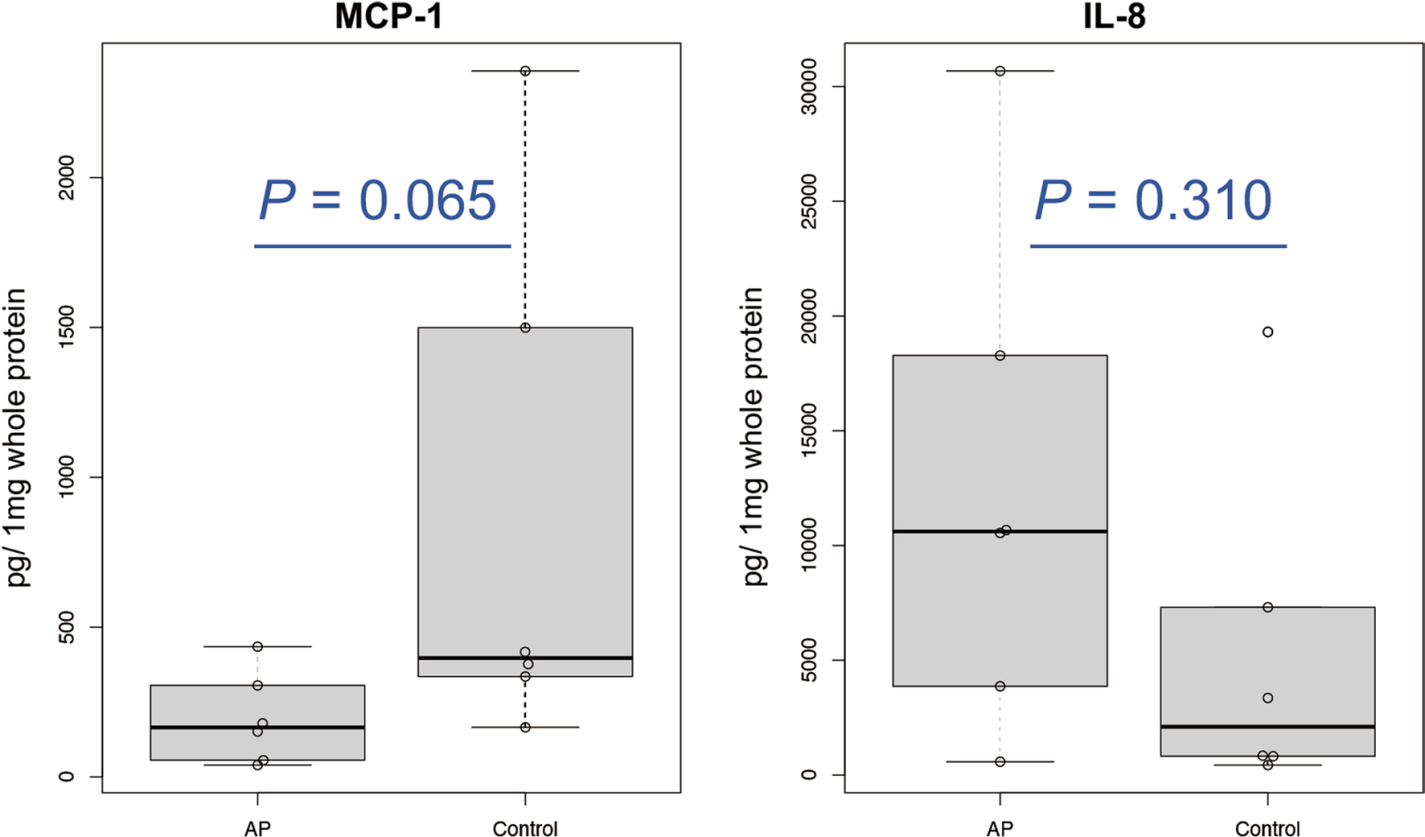
Results of the multiplex cytokines/chemokines assay. *P* values were calculated by the Mann–Whitney U test

In our shotgun proteomics analysis, we did not detect peaks for IL-1β, IL-6, TNF-α, or MCP-1 (Additional file 1: Table S2). In the multiplex cytokine/chemokine assay, the values for IL-6 and TNF-α were under the limit of detection (IL-6, < 6.80 pg/mL; TNF-α, < 0.73 pg/mL) in most of the oral samples. Using the supernatant without TCA precipitation, we also conducted a multiplex cytokine/chemokine assay. The protein concentrations of the supernatant ranged from 0.012 to 0.27 mg/mL, which were likely dependent on the strength of rubbing. Among the 13 cytokine and chemokine proteins, IL-1β, MCP-1, IL-8, and IL-18 were detected in all 12 samples. The concentrations were normalized to the total protein concentration. There were no significant differences between the AP and control groups, although MCP-1 levels tended to be lower (*P* = 0.065 by Mann–Whitney U test; Fig. 3).

## Discussion

Underlying diseases can affect the composition of oral proteins. Figura et al. reported lower concentrations of S100-A16, ARP2/3, and VPS4B in the saliva of the Parkinson’s disease group compared with the healthy control group [30]. Although the three proteins were detected with high confidence in the present study, there was no significant difference between the three Parkinson’s disease samples (AP#3, AP#5, and AP#6) and the other nine samples. Concerning Alzheimer's disease, Contini et al. reported higher levels of S100A8, S100A9, α-defensins, and cystatins A and B in patients with Alzheimer's disease compared with healthy volunteers [31]. Although our analysis detected α-defensin, there was no significant difference between the three samples from Alzheimer's disease patients (AP#2, AP#4, and Control#1) and the other nine samples. These data suggest that the changes in the protein concentration disappear in patients suffering from AP. Absence of significant difference between subsets may be attributed to the limited size of our cohort study.

The S100 proteins, a family of calcium-binding cytosolic proteins, are known as damage-associated molecular pattern molecules and exhibit a variety of intracellular and extracellular functions [25]. The S100 protein family consists of approximately 20 members, which are not only involved in cell proliferation, differentiation, migration, and apoptosis but are also thought to be closely related to cancer and neurodegenerative diseases [32]. Of these, S100-A7 is abundant in the saliva of patients with systemic sclerosis [33] and has recently been reported to act as an antimicrobial peptide [34, 35]. The S100-A7 is produced in [36] epithelial cells on the tongue and has been shown to exhibit antimicrobial activity against *Escherichia coli* (*E. coli*) [37]. The S100-A7A may be a useful biomarker for AP.

Human serpins are a family of endogenous protease inhibitors with several biological functions [38]. As Bao et al. reviewed, serpin family proteins are involved in host–pathogen interactions [39]. Jiang et al. reported that α-antitrypsin, a serpin superfamily member, promotes lung defense against *Pseudomonas aeruginosa* by inhibiting neutrophil elastase-mediated host defense protein degradation in mice [40]. Moreover, serpin A1 suppresses the mediators of lipopolysaccharide-mediated proinflammation [41, 42]. Association of serpin 4B to immunity and/or infection remains unclear.

Peptidoglycan is an essential component of the bacterial cell envelope [43]. Peptidoglycan recognition proteins recognize bacterial peptidoglycans and are involved in promoting antibacterial immunity and inflammation [44]. For example, human peptidoglycan recognition protein 1 exhibits bactericidal activity and is found in body fluids such as serum, sweat, and saliva [45]. In this study, we observed a high abundance of peptidoglycan recognition protein 1 (*P* = 0.093 by Mann–Whitney U test) (AP/Control ratio = 2.6, Additional file 1: Table S2). In addition, its abundance showed a significantly negative correlation with the time post-onset (*P* = 0.042 by Spearman’s test, Additional file 1: Table S4). Based on our data and previous reports, peptidoglycan recognition protein 1 and protein S100A-7A may be useful biomarkers of AP.

Cytokines and chemokines are known markers of inflammation in response to bacterial infection. Although MCP-1 is a chemokine that recruits monocytes to the foci of active inflammation [46, 47], it was detected in all samples by the multiplex assay and the values tended to be lower in the AP group compared with the control group (Fig. 3). McGrath-Morrow et al. showed that in a lower respiratory tract model of *E. coli* infection, the host defense against the bacterium was mediated by MCP-1 and its receptor, CCR2 [47]. Low MCP-1 levels in AP patients may explain the reduced resistance to infection.

## Limitations

As mentioned in Introduction, it remains unclear whether AP is distinct from typical pneumonia [1, 10, 11]. To clarify this, patients with typical pneumonia should be recruited as controls and compared with those having AP. However, in the current study, we could not recruit such patients as controls, suggesting that the potential biomarkers discovered in this study are not specific to AP in cases where there is any difference between AP and typical pneumonia.

## Conclusion

In this study, we identified putative biomarkers applicable to the detection of AP at an early stage. We found four candidate proteins that may be considered biomarkers of AP. This study had several limitations, which included the varied duration from onset to collection (1–18 days post-onset). It remains unclear whether the candidate proteins identified in this study increase or decrease in the early stages of the disease. To address this issue, long-term prospective studies need to be conducted that evaluate samples from pre-onset to the onset of AP.

## Supplementary Information

**Additional file 1: Table S1.** Characteristics of the 12 patients. **Table S2.** Detected Proteins. (P values were calculated by ANOVA and adjusted by the Benjamini–Hochberg method in Proteome Discoverer software version 2.2.0.388 (Thermo Fisher Scientific).). **Table S3.** 602 proteins with high confidence (FDR) and high coverages (>50%). (P values were calculated using protein abundance (columns F-Q) by Mann–Whitney U test.). **Table S4.** Correlation between days post-onset and the proteins. (P values were calculated by Spearman's test).

**Additional file 2: Figure S1.** Distribution of abundance. This figure was prepared by Proteome Discoverer software version 2.2.0.388 (Thermo Fisher Scientific). **Figure S2. **Volcano plots of the proteomic data. The plots were generated using the abundance ratio = Log2(AP/Control). *P* values were calculated by ANOVA and adjusted by the Benjamini–Hochberg method in Proteome Discoverer software version 2.2.0.388 (Thermo Fisher Scientific). **Figure S3.** Principal component analysis of the 12 samples. This figure was prepared by Proteome Discoverer software version 2.2.0.388 (Thermo Fisher Scientific).

## Data Availability

The LC–MS/MS data are described in Additional file 1: Table S2. The raw data of the 12 samples are deposited in the jPOST Repository/Database (Proteome Xchange Accession number PXD037636) [48].

## Abbreviations

AP: Aspiration pneumonia
CRP: C-reactive protein
LC–MS/MS: Nanoliquid chromatography and tandem mass spectrometry
