# Supplementary material for: Potential biomarker proteins for aspiration pneumonia detected by shotgun proteomics using buccal mucosa samples: a cross-sectional case–control study
Source: Clin Proteomics. 2023 Mar 9;20:9. doi: 10.1186/s12014-023-09398-w (PMC9996945; doi:10.1186/s12014-023-09398-w)
Supplement: Supplementary file 2 — Additional file 2: Figure S1. Distribution of abundance. This figure was prepared by Proteome Discoverer software version 2.2.0.388 (Thermo Fisher Scientific). Figure S2. Volcano plots of the proteomic data. The plots were generated using the abundance ratio = Log2(AP/Control). P values were calculated by ANOVA and adjusted by the Benjamini–Hochberg method in Proteome Discoverer software version 2.2.0.388 (Thermo Fisher Scientific). Figure S3. Principal component analysis of the 12 samples. This figure was prepared by Proteome Discoverer software version 2.2.0.388 (Thermo Fisher Scientific). [file 12014_2023_9398_MOESM2_ESM.pdf]

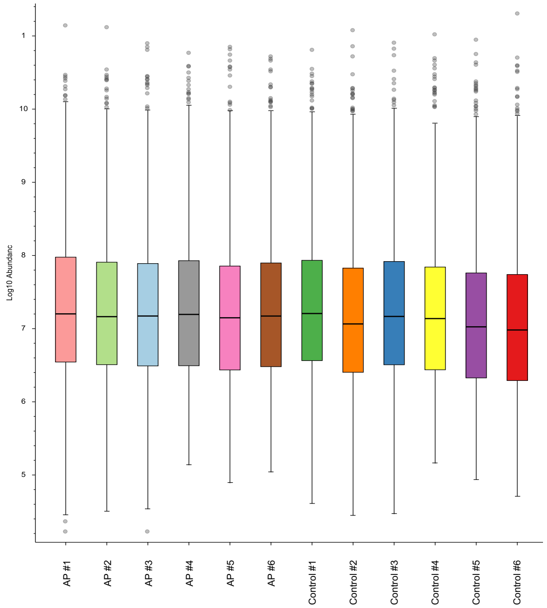

Figure S1. Distribution of abundance. This figure was prepared by Proteome Discoverer software version 2.2.0.388 (Thermo Fisher Scientific).

Supplementary Materials
Ogura et al.
Potential biomarker proteins for aspiration pneumonia detected by shotgun proteomics using buccal mucosa samples: A cross-sectional case–control study

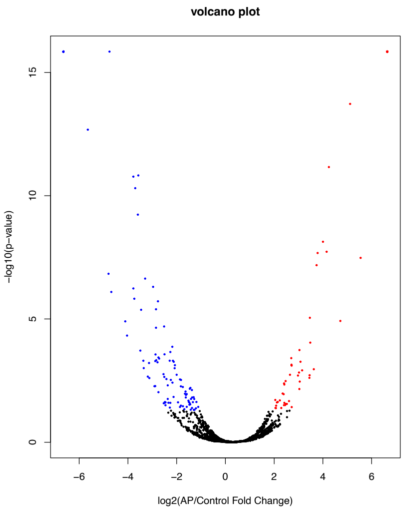

Figure S2. Volcano plots of the proteomic data. The plots were generated using the abundance ratio = Log2(AP/Control). *P* values were calculated by ANOVA and adjusted by the Benjamini–Hochberg method in Proteome Discoverer software version 2.2.0.388 (Thermo Fisher Scientific).

Supplementary Materials

Ogura et al.

Potential biomarker proteins for aspiration pneumonia detected by shotgun proteomics using buccal mucosa samples: A cross-sectional case–control study

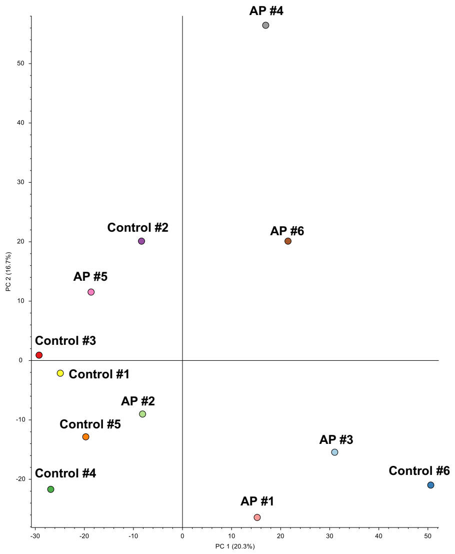

Figure S3. Principal component analysis of the 12 samples. This figure was prepared by Proteome Discoverer software version 2.2.0.388 (Thermo Fisher Scientific).
